# Association between body fat composition and blood pressure level among secondary school adolescents in Dar es Salaam, Tanzania

**DOI:** 10.11604/pamj.2014.19.327.5222

**Published:** 2014-11-27

**Authors:** Brighton Mushengezi, Pilly Chillo

**Affiliations:** 1 Muhimbili University of Health and Allied Sciences, Dar es Salaam, Tanzania

**Keywords:** hypertension, adolescents, body fat percent

## Abstract

**Introduction:**

Excess body fat and high blood pressure (BP) are important risk factors for increased cardiovascular morbidity and mortality, and both may have their roots of occurrence in childhood and adolescence. The present study aimed at determining the association between body fat composition and BP level among adolescents in Tanzania.

**Methods:**

A cross-sectional study involving 5 randomly selected secondary schools within Dar es Salaam was conducted between June and November 2013. Structured questionnaires were used to collect information on demographic characteristics and other cardiovascular risk factors. BP, height, weight and waist circumference were measured following standard methods. Body fat was assessed by skinfold thickness and categorized as underfat, healthy, overfat or obese according to World Health Organization definitions. Hypertension was defined as BP ≥ 90^th^ percentile for age, height and gender of the adolescent.

**Results:**

The study included 582 adolescents (mean age 16.5±1.8 years, 52.1% boys). The proportion of adolescents with overfat or obesity was 22.2%. Systolic, diastolic and combined hypertension was present in 17.5%, 5.5%, and 4.0% respectively. In the total population mean body fat percent correlated positively with diastolic BP and mean arterial pressure (MAP) but not with systolic BP. In multivariate analysis body mass index (β=0.21, p=0.008) and waist circumference (β=0.12, p=0.049), but not body fat percentage (β=-0.09, p=0.399) independently predicted higher MAP.

**Conclusion:**

Body mass index predicts BP level better than body fat composition and should be used as a measure of increased risk for hypertension among adolescents.

## Introduction

Hypertension and excess body weight are major cardiovascular risk factors and are a cause of concern in many parts of sub Saharan Africa due to the high prevalence, especially in urban populations [1]. Among adults, hypertension and excess body weight are closely related and overweight and obese individuals are more likely to be hypertensive [2]. Moreover, blood pressure decreases in a dose-response fashion when weight is reduced [3]. Both hypertension and excess body weight may have their root of occurrence in childhood and adolescence, and overweight adolescents are more likely to become overweight adults [4]. Worldwide, the prevalence of overweight and obesity among children and adolescents has shown a remarkable increase [5–7], not only in developed but also in sub Saharan African countries [8]. Although the prevalence of hypertension is still low among children and adolescents in the region, recent studies have shown increased trends, with hypertension likely to occur among those with overweight and obesity [9].

Having the world's highest prevalence of hypertension among adults [10], sub Saharan Africa must focus on primary prevention as an important intervention. Therefore a better understanding of the precursors of hypertension is of great significance. Although body mass index (BMI) has been widely used to measure overweight and obesity, it does not however distinguish between fat and lean body mass and it is the amount of body fatness that is associated with morbidities including hypertension, more strongly so than lean body weight [11]. Skinfold thickness is a cheap and easy way to measure body fatness and can be applied as a tool to screen for excess body fat. It measures subcutaneous fat located directly beneath the skin by grasping a fold of skin and subcutaneous fat and measuring its thickness using calipers. Formulas then take account of age and sex and convert skinfold measurements into an estimate of an individual's percentage body fat [12]. Ideally, body fat percent should correlate better with BP and may be a better way to assess and track excess body weight among children and adolescents. However, it is not known in Tanzania if increase in body fatness is a better estimate of BP level than BMI. The present study was therefore set out to determine the association between body fat percent and BP levels among adolescents and to determine if body fat percentage is superior to BMI and waist circumference in estimating BP among adolescents in Tanzania.

## Methods

### Study design and population

This was a cross-sectional study of secondary school adolescents aged 12 to 19 years in Dar es Salaam, Tanzania. A minimum sample size of 523 adolescents was needed to determine the association between overweight and obesity with BP levels with a maximum likely error of 2.5% at 95% confidence interval.

### Sampling procedure

A list of all secondary schools in Dar es Salaam was obtained from the Ministry of education and Social Welfare. Five schools were randomly selected from this list. A multistage sampling procedure was then performed to obtain the required sample size.

### Data collection

All selected participants received information about the study and were asked for their willingness to participate. All agreeing participants had to sign an informed consent form before data collection. A structured questionnaire was used to obtain demographic characteristics as well as to obtain history on cardiovascular risk factors. Height was measured using a stadiometer (Seca, CEO123, and USA), with subjects wearing no shoes and averaged to the nearest centimeter. Weight was measured by a weighing scale (Momert, China) and recorded in kilogram. BMI was calculated as weight in kilogram divided by height in meter squared. Obesity and overweight were defined as BMI ≥30kg/m^2^ and ≥25 kg/m^2^ respectively for adolescents aged 18 years and above. For adolescents who were less than 18 years, overweight and obesity was defined according to WHO cut-off points for adolescents [13]. Waist circumference was measured using a tape measure mid way between the anterior superior iliac spine and the lowest rib and was recorded to the nearest centimeter.

Blood pressure was taken using a digital BP machine (OMRON CEO 197, Kyoto, Japan.). Measurements were done in a quiet room after a 5 minutes rest. A total of three readings were taken, 5 minutes apart. The average of the last two readings was taken as the subject's BP. Hypertension was defined as blood pressure ≥140 and /or 90mmHg for adolescents who were ≥18 years. For adolescents aged less than 18 years, hypertension was defined as BP ≥90^th^percentile for age, height and gender of the adolescent [14]. Mean arterial pressure (MAP) was calculated using the formula; MAP = Diastolic BP + 1/3(Systolic BP-Diastolic BP). Skin fold thickness was obtained using a skin fold caliper (Harpenden Skinfold Caliper, Barty International, CE 120, England) at standardized sites for boys and girls. Measurements were done at 7 sites, including triceps, pectoral, axillary, abdominal, subscapular, suprailiac and thigh. All skinfold measurements were taken by the same individual (BM). Measurements were taken on the right side of the body and recorded in millimeters. The skinfold was picked between the thumb and the index finger so as to include two thicknesses of the skin and subcutaneous fat. Calipers were located about one centimeter from the finger with the calipers halfway up the fold of the skin. Measurements were done twice and an average value was recorded. The sum of three skinfold measurements was used to calculate body density, according to Jackson and Pollock equation [15, 16]. For girls, triceps, suprailiac and thigh skinfold measurements were used and for boys pectoral, abdominal and thigh skinfolds were used. Body density was calculated as follows; For girls: Body Density = 1.0994921 - 0.0009929*sum + 0.0000023*sum2 - 0.0001392*age. For boys: Body Density = 1.1093800 - 0.0008267*sum + 0.0000016*sum2 - 0.0002574*age. Where sum = sum of three skinfold thicknesses as mentioned above.

After obtaining the body density, body fat percent was estimated using the Siri formula [17] as follows; Percent fat (%) = ((495 / Body Density) -450) * 100. Increased percent fat was defined as overfat and obese which is >85^th^ percentile and >95^th^ percentile respectively according to specific charts for age and sex of the adolescent [18].

### Statistical Methods

Data entry and analysis was done using Statistical Package for Social Sciences (SPSS) version 20. Descriptive statistics are presented as mean ±SD for continuous variables and percentages for categorical variables. Groups were compared using Chi square test, Student's t-test, or Analysis of Variance (ANOVA) as appropriate. Univariate analysis was done using Pearson's correlation coefficient. Multivariate linear regression analysis was used to determine the independent predictors of MAP. A two-sided p-value of <0.05 was considered statistically significant.

### Ethical issue

Ethical clearance was obtained from the Research and Publication Committee of Muhimbili University of Health and Allied Sciences (MUHAS). Subjects 18 years and above signed an informed consent form. For minors (17 years and below), parents/guardians signed on their behalf. Permission was also sought from the heads of schools.

## Results

The study population included 582 (303 boys and 279 girls) adolescents. The mean ±SD age of the total population was 16.5 ±1.8 years, with boys being on average 0.7 years older than girls, Table 1. Significantly more girls (78.1%) were mostly sedentary during their spare time when compared to boys (44.9%), p<0.001. Girls were also more likely to have a positive family history of hypertension (15.1% versus 8.9%), while boys were more likely to be exposed to passive smoking (16.8% versus 11.1%), all p<0.05. On average boys were taller than girls, (164.8 ± 9.0cm versus 157.0 ± 6.0 cm), p<0.001; however there was no significant difference in the mean weight among boys and girls, Table 1. When compared to boys, girls had significantly higher mean values for BMI as well as waist circumference and they were more likely to be overweight and obese, all p<0.01, Table 1. The mean ±SD systolic and diastolic BP of the total population was 120 ±11 mmHg and 69 ±8 mmHg, respectively. Boys had higher mean values of systolic BP (121 ±11 versus 118 ±10mmHg), however there was no difference between boys and girls in terms of the proportions with systolic hypertension, Table 1.

**Table 1:** Demographic and anthropometric measurements by gender

Characteristic	Boys (N = 303)	Girls (N = 279)	*p*-value
Age (years)	16.9 ± 1.7	16.2 ± 1.9	<0.001
Height (cm)	164.8 ± 9.0	157.0 ± 6.0	<0.001
Weight (kg)	56.3 ± 8.7	55.0 ± 10.4	0.86
Body mass index (kg/m^2^)	20.8 ± 3.1	22.3 ± 3.9	<0.001
Proportion with overweight and obesity n(%)	40 (13.2)	61 (21.9)	0.006
Waist circumference (cm)	73 ± 8	76 ± 11	0.001
Positive family history of hypertension n (%)	27 (8.9)	42 (15.1)	0.022
Proportion exposed to passive smoking n (%)	51 (16.8)	31 (11.1)	0.048
Systolic blood pressure (mmHg)	121 ± 11	118 ± 10	0.002
Proportion with systolic hypertension n (%)	46 (15.2)	56 (20.1)	0.121
Diastolic blood pressure (mmHg)	68 ± 8	71 ± 8	<0.001
Proportion with diastolic hypertension n (%)	11 (3.6)	21 (7.5)	0.039
Mean arterial pressure (mmHg)	86 ± 8	87 ± 8	0.107
Proportion with systolic and diastolic hypertension n (%)	8 (2.6)	15 (5.4)	0.091

The mean diastolic BP was higher in girls (71 ±8 versus 68 ± 8mmHg), and significantly more girls had diastolic hypertension when compared to boys (7.5% versus 3.6%) p<0.05 for both, Table 1. Twenty three (4%) adolescents had a combination of both systolic and diastolic hypertension. The proportions of adolescents with underweight, healthy, overweight and obesity by BMI categorization was 13.9%, 72.9%, 12.5% and 0.7% respectively among boys, and 5.4%, 72.8%, 14.3% and 7.5% respectively among girls. This difference was statistically significant, p<0.001. Girls had higher mean values for skinfold measurements in all the seven sites when compared to boys, Table 2. Consequently, the mean sum of skinfold thickness was significantly higher for girls (76.8 ±27.2 versus 42.2 ± 22.4mm), p<0.001, Table 2. The mean total body fat percent for girls was more than twice that of boys (27.8 ± 6.7% versus 11.1 ± 6.2%) and significantly more girls had increased total body fat percent (32.6% versus 12.5%), all p<0.001, Table 2. The proportion of adolescents with underfat, healthy, overfat and obesity by fat percentage categorization was 62.7%, 24.8%, 7.6% and 5.0% respectively among boys and 3.6%, 63.8%, 17.6% and 15.1% respectively among girls. This difference was statistically significant, p<0.001.

**Table 2:** Body fat composition by gender

Characteristic	Male (N = 303)	Female (N = 279)	*p*-value
Triceps skinfold thickness (mm)	12.7 ± 7.4	21.9 ± 6.8	<0.001
Subscapular skinfold thickness (mm)	13.3 ± 5.9	19.6 ± 7.6	<0.001
Axillary skinfold thickness (mm)	12.0 ± 6.3	21.0 ± 9.1	<0.001
Pectoral skinfold thickness (mm)	9.8 ± 5.5	17.9 ± 6.9	<0.001
Abdominal skinfold thickness (mm)	16.4 ± 8.6	27.9 ± 9.5	<0.001
Suprailiac skinfold thickness (mm)	12.7 ± 7.3	21.1 ± 7.9	<0.001
Thigh skinfold thickness (mm)	16.1 ± 9.8	32.7 ± 10.6	<0.001
Sum of skinfold thickness (mm)	42.2 ± 22.4	76.8 ± 27.2	<0.001
Body density (g/cm^3^)	1.074 ± 0.014	1.036 ± 0.015	<0.001
Total body fat percent (%)	11.1 ± 6.2	27.8 ± 6.7	<0.001
Proportion with overfat and obesity n (%)	38 (12.5)	91 (32.6)	<0.001

In the total population, increase in the mean systolic BP was associated with increase in age, height, weight, body mass index, waist as well as the hip circumference, with weight showing the best correlation (r = 0.37, p<0.001), Table 3. Measures of skinfold thickness at all sites as well as the mean sum of skinfold thickness did not show any significant correlation with mean systolic BP in the total population, Table 3. When analysis was separated for boys and girls, all the conventional measures of adiposity (age, height, weight, BMI, waist circumference, hip circumference) correlated positively with increase in mean systolic BP, again weight of the individual showing the best correlation, both in boys (r = 0.42, p<0.001) and in girls (r = 0.32, p<0.001), Table 3. Sub scapular, axillary and abdomen skinfold thicknesses had weak, but positive and significant correlations with mean systolic BP both in boys and girls separately. In addition, among girls supra iliac and thigh skinfolds thicknesses significantly correlated with mean systolic BP. The mean sum of skinfold thicknesses and body fat percent positively correlated with increase in mean systolic BP in girls alone, Table 3.

**Table 3:** Correlates of systolic blood pressure, diastolic blood pressure and mean arterial pressure in the total population and in boys and girls

	SBP (mmHg)			DBP (mmHg)			MAP (mmHg)		
Variables	Total	Boys	Girls	Total	Boys	Girls	Total	Boys	Girls
	Pearson's correlation	Pearson's correlation	Pearson's correlation	Pearson's correlation	Pearson's correlation	Pearson's correlation	Pearson's correlation	Pearson's correlation	Pearson's correlation
Age (years)	0.14*	0.16*	0.08 ^NS^	-0.01 ^NS^	0.02 ^NS^	0.03 ^NS^	0.05^NS^	0.08^NS^	0.05^NS^
Height (cm)	0.23**	0.25**	0.12*	0.01 ^NS^	0.09 ^NS^	0.11 ^NS^	0.11*	0.17*	0.12*
Weight (kg)	0.37**	0.42**	0.32**	0.23**	0.27**	0.23**	0.32**	0.36**	0.29**
BMI (kg/m^2^)	0.24**	0.26**	0.29**	0.24**	0.22**	0.20*	0.26**	0.26**	0.26**
Waist circumference (cm)	0.18**	0.28**	0.14*	0.22**	0.31**	0.13*	0.25**	0.33**	0.18*
Triceps (mm)	-0.04 ^NS^	0.002 ^NS^	0.09 ^NS^	0.19**	0.13*	0.09 ^NS^	0.11*	0.09^NS^	0.10^NS^
Subscapular (mm)	0.07 ^NS^	0.11*	0.17*	0.23**	0.20*	0.15*	0.19**	0.18*	0.17*
Axillary (mm)	0.06 ^NS^	0.12*	0.16*	0.23**	0.18*	0.15*	0.18**	0.17*	0.17*
Pectoral (mm)	-0.03 ^NS^	0.05 ^NS^	0.06 ^NS^	0.24**	0.18*	0.18*	0.15**	0.14*	0.14*
Abdomen (mm)	0.04 ^NS^	0.12*	0.15*	0.24**	0.21**	0.13*	0.18**	0.20*	0.15*
Suprailliac (mm)	0.04 ^NS^	0.10 ^NS^	0.14*	0.19**	0.15*	0.09 ^NS^	0.15**	0.14*	0.12*
Thigh (mm)	0.01 ^NS^	0.05 ^NS^	0.19*	0.24**	0.17*	0.17*	0.17**	0.14*	0.19*
Sum skin (mm)	0.02 ^NS^	0.08 ^NS^	0.15*	0.23**	0.20*	0.12*	0.16**	0.17*	0.14*
Body density (g/cm^3^)	0.03 ^NS^	-0.09 ^NS^	-0.18*	-0.25**	-0.19*	-0.14*	-0.16**	-0.17*	-0.17*
Body fat percent (%)	-0.02 ^NS^	0.09 ^NS^	0.18*	0.25**	0.20*	0.14*	0.16**	0.17*	0.17*

Increase in mean diastolic BP was associated with increase in weight, BMI as well as waist circumference in the total population and when analysis was separated for boys and girls, Table 3. Interestingly, age and height did not show any relationship with mean diastolic BP either in the total population or in boys and girls alone. Contrary to findings with mean systolic BP, measures of adiposity by skinfold measurement showed fairly good correlations with mean diastolic BP in the total population as well as in boys and girls, with all skinfold thicknesses at the seven sites showing significant and positive correlations with mean diastolic BP in the total population and in boys. Among girls, only sub scapular, axillary, pectoral, abdomen and thigh skinfold thicknesses positively correlated with mean diastolic BP, Table 3. Both mean sum of skinfold thickness and body fat percentage positively and significantly correlated with mean diastolic BP in the total population and in boys and girls separately, Table 3.

**Figure 1:**
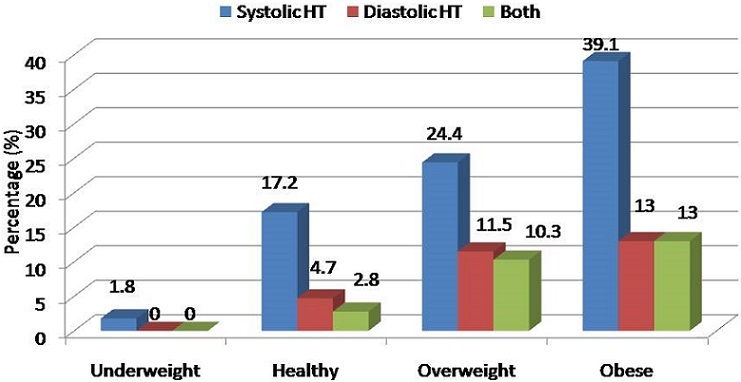
Prevalence of systolic, diastolic and both hypertension in underweight, healthy, overweight and obese adolescents according to body mass index

Except for age, the rest of the measures of adiposity correlated positively and significantly with MAP in the total population (all p<0.05). When analysis was done separately; age and triceps skinfold thickness did not correlate with MAP in boys and girls. Other measures of adiposity correlated positively and significantly with MAP both in boys and girls (all p<0.05), Table 3. In the total population, the prevalence of systolic and diastolic hypertension increased in a step-wise fashion with increase in BMI. The prevalence of systolic hypertension was 1.8%, 17.2%, 24.4% and 39.1% among adolescents who were underweight, healthy, overweight and obese, respectively, p<0.001, Figure 1. Diastolic hypertension showed the same trend, and it was found in 0%, 4.7%, 11.5% and 13% among underweight, healthy, overweight and obese adolescents respectively, p<0.001, Figure 1.

**Figure 2:**
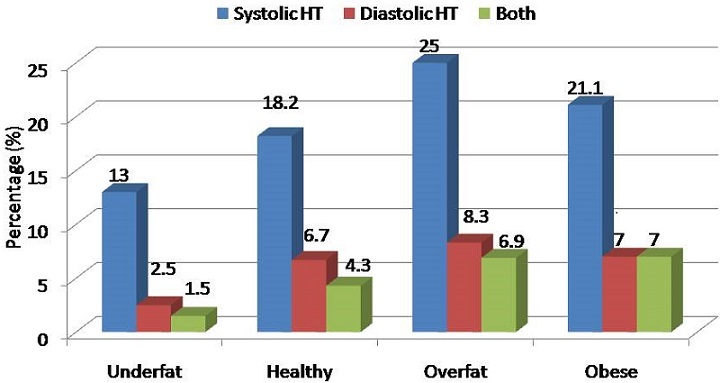
Prevalence of systolic, diastolic and both hypertension in underfat, healthy, overfat and obese adolescents according to body fat percentage

When body fat percent was used to categorize adolescents into underfat, healthy, overweight and obese groups, there was generally a trend towards increase in the prevalence of both systolic and diastolic hypertension. However, the fat percentage categorization could not differentiate those with overweight and obesity in terms of the prevalences of both systolic and diastolic hypertension. Systolic and diastolic hypertension increased from underfat, to healthy and to overweight adolescents and slightly but not significantly decreased in those who were obese, Figure 2. In multivariate linear regression analysis, higher MAP was independently associated with higher BMI (β = 0.21, p = 0.008) and higher waist circumference (β = 0.12, p = 0.049), but not with higher body fat percent. These associations were independent of age and gender of the adolescent, Table 4.

**Table 4:** Independent predictors of higher mean arterial pressure in the total population identified in multivariate linear regression analysis

Variable	Beta coefficient (β)	t	p-value
Age (years)	0.033	0.804	0.422
Gender (boys vs girls)	-0.081	-1.006	0.315
Waist circumference (cm)	0.120	1.962	0.049
Body mass index (kg/m^2^)	0.206	2.657	0.008
Body fat percent (%)	-0.085	-0.845	0.399

## Discussion

Hypertension and other cardiovascular diseases are on the increase in sub Saharan Africa and estimates show that by 2030 cardiovascular diseases will bypass communicable disease including tuberculosis and malaria as the most important cause of morbidity and mortality in the region [19]. Understanding the roots of hypertension is therefore of major public health importance as a step towards primary intervention. One area that may be of interest is to learn the occurrence of hypertension from adolescence. The present study found the proportion of adolescents with systolic and diastolic hypertension to be 17.5% and 5.5% respectively, while 4% of the total studied had both systolic and diastolic hypertension. These results are similar to those found in Nigeria among 9-18 year olds in Lagos [20] and among 10-18 year olds in Enugu adolescents [21]. The findings are however different from those found in urban school children in Chennai, India where the prevalences were higher at 21.5% for both systolic and diastolic hypertension [22].

Previous studies in Tanzanian adolescents found the prevalence for hypertension to be much lower [23] and this difference could be explained by the general observations that hypertension prevalence is on the increase in Tanzania. The difference could also be due to the differences in the population studied. In the study by Kitange et al, [23] adolescents were from rural and semi-urban areas, partly explaining the lower prevalences in their cohort as hypertension is known to be more prevalent among urban dwellers. In that study which took place way back in 1980's combined systolic and diastolic hypertension was found only in 0.4%. Although the study by Chillo et al [24] included a much younger age group (6- 15years old), the prevalence of systolic, diastolic and both hypertension was almost similar to the present study among 12-19 year old adolescents. As also noted in the literature the prevalence of systolic hypertension was 11.4%, that of diastolic hypertension was 8.1% and 3.9% had both systolic and diastolic hypertension in the study by Chillo, et al. This similarity can be explained by the similarities in the population studied, as both studies were in urban settings.

This study found that mean systolic blood pressure was greater in adolescent boys than girls while the mean diastolic blood pressure was greater in adolescent girls than boys. Moreover, girls were more likely to have diastolic hypertension. This finding is not unique to this study, as Fitzpatrick et al had similar findings among African American adolescents [25] and Ujunwa et al found similar pattern among adolescent Nigerians attending secondary schools in Enugu metropolis [21]. The finding in this study could be explained by the observation that girls had higher BMI as well as higher body fat percentage and were more likely to be overweight and obese when compared to boys. As shown in the findings, diastolic blood pressure was much more associated with body fat percent than systolic blood pressure and hence the difference between boys and girls. Girls were also more likely to report a family history of hypertension and generally girls were mostly sedentary. Both family history of hypertension and sedentary lifestyle are known risk factors for hypertension.

Apart from observed findings from this study, several other reasons could explain blood pressure gender differences in this population. In the study by Ujunwa et al [21], findings similar to this study were noted among Nigerian adolescents, hormonal changes occurring at puberty being attributed to the observed differences. The psychosocial stress at menarche and rapid physiological changes that accelerate completion of puberty among girls have been documented to be associated with an increase in blood pressure among adolescent girls [21].

The relationship between body composition and BP levels has well been established in epidemiological studies, and in adolescents and childhood BP is positively correlated with age, weight, height as well as height/weight measurements [20, 26]. Traditionally, body composition has been estimated by BMI as well as waist circumference and these have been shown to positively correlate with levels of BP. This study found height, weight, BMI and waist circumference to positively and significantly correlate with both systolic and diastolic BP. As an alternative and may be more appropriate measure of adiposity, body fat percentage ideally would have been the best estimate of excess fat and therefore would have correlated better with BP levels. In the present study, we found percent body fat to correlate with diastolic but not systolic BP in the total population and when compared to the conventional body build measurements, it was not better. Even when different sites were considered differently, the correlates were rather weak when compared to BMI. Furthermore, in multivariate analysis body fat percent was not independently associated with MAP, which is a measure that takes account the systolic and diastolic BP of the individual. These findings have been reported by other previous researchers [6, 27]. It is possible that body fat percentage is the best measure among adults, as in adolescents and children subcutaneous fat may be a normal growing pattern; especially in girls and BP differences are actually differences in body build and normal growth patterns. Although inaccurate skinfold measurement could have contributed to the poor or lack of association between body fat percentage and BP, this factor if present would be negligible as all measurements were done by the same person and in pre-specified anatomical sites.

The finding that there was a big difference in the proportion of underfat and underweight by the fat percentage and BMI categorization warrants some explanation. In this study, the proportion of underfat adolescents in boys was alarming, being 62.2%. This finding could have been a true picture and indicates that many adolescents are otherwise underfat and therefore at an increased risk of conditions of malnutrition and stunting. On the other hand, this could be just an over-estimation of the underfat category as with the conventional BMI, the proportion of underweight was in the contrary not alarming, again giving an indication that the best measure of body builds in this population is BMI. Widiyani et al found the proportion of adolescents with underfat to be 12.5% using the same cut-off points [28], therefore suggesting that the present results could as well be a true picture of the state of the adolescents in this study population and maybe in the country, underscoring the importance of looking both into overweight and obesity end as well as the lower end of underfat as it is also not without disease risk including future cardiovascular and renal diseases.

One of the most important finding of this study is that BMI categorization into underweight, healthy, overweight and obesity was associated with a clear step-wise increase in the proportion of adolescents with both systolic and diastolic hypertension. Among underweight adolescents for example, systolic hypertension was present in only 1.8% while it was present in 39.1% in those who were obese. This pattern was not however the same with body fat percent, further showing the superiority of BMI over fat percentage in relation to BP and hypertension in this study population. Use of skinfold measurement on estimating body fat percent, instead of more accurate methods like dual energy x-ray absorptiometry or bioelectric impedance analysis may have contributed to differences in association between body fat percent and BP. However skinfold thickness being one of the validated methods of measuring body fat composition is comparatively cheaper and can easily be applied in clinical settings.

## Conclusion

Body fat percentage measured by skinfold thickness method did not predict blood pressure level better than BMI in this population. High BMI should be used as a measure of increased risk for hypertension among adolescents.
